# Correlation between dental conditions and comorbidities in an elderly Japanese population: A cross-sectional study

**DOI:** 10.1097/MD.0000000000011075

**Published:** 2018-06-15

**Authors:** Kazuki Ide, Kahori Seto, Tomoko Usui, Sachiko Tanaka, Koji Kawakami

**Affiliations:** a Department of Pharmacoepidemiology, Graduate School of Medicine and Public Health; b Center for the Promotion of Interdisciplinary Education and Research, Kyoto University, Kyoto; c Department of Medical Statistics, Shiga University of Medical Science, Seta Tsukinowa-cho, Otsu, Shiga, Japan.

**Keywords:** database, nursing home, older adults, oral health

## Abstract

The purpose of this study was to investigate the correlation between dental conditions and comorbidities in an elderly population in Japan.

A database constructed using data obtained from 12 nursing homes in Japan was used in this study. The study period ranged from January 2014 to December 2015, and elderly individuals with dental and other medical records were included in the analysis. Linear regression models were used to analyze univariate and multivariate correlation between dental conditions, comorbidities, and other sociodemographic/clinical backgrounds.

After excluding individuals with missing data, 289 elderly individuals (107 men and 182 women; mean age, 85 years) were included in the analysis. These individuals had an average of 11.6 teeth, and the number decreased with older age (*P* < .001). The average number of decayed teeth was 1.4. A total of 116 subjects (40.1%) had dementia. In terms of comorbidities, dementia was not significantly associated with the number of present teeth (*P* = .56), but it was associated with the number of decayed teeth (*P* = .018). This association was also observed after adjusting for confounding variables in the multivariate regression analysis (*P* = .030).

The number of decayed teeth was associated with dementia. While causality cannot be inferred from these observational results, the findings indicate that dental health could represent a marker of impending dementia, and probably represent a marker of general health status in the elderly population. Additional longitudinal studies are highly desirable to understand the causal relationships between dental conditions and comorbidities.

## Abstract 2

Supplemental Digital Content is available in the text

## 1 Introduction

Japan is one of the countries that contain the most rapidly aging populations in the world; the percentage of persons aged ≥65 years reached 27.3% in 2017.^[1,2]^ The percentage is estimated to exceed 30% by 2025. However, this trend can now be observed globally.^[3]^ Most people can expect to live over 60 years, and this also occurs in less developed countries.^[4,5]^ This is the first time in history mankind has been dealing with this issue, and thus, medical and preventive care for the elderly population is becoming increasingly important.^[4,6]^

One aspect of health care that is particularly important in the elderly is oral care.^[7]^ Tooth loss can give rise to various problems associated with eating, speaking, and appearance.^[8]^ The number of teeth or wearing of dentures is also related to swallowing function.^[9]^ Moreover, hypertension,^[10]^ cardiovascular disease,^[11–13]^ and oral/dental conditions due to poor oral health have been reported in several population groups. Dental conditions are also associated with diet and nutritional status.^[14]^ Therefore, oral/dental health problems could affect general health and quality of life both directly and indirectly.

Previous research has shown that older adults who need special care are at an increased risk for dental plaque accumulation on natural teeth or dentures. A higher incidence of caries, gingivitis, periodontal disease, and edentulousness is also observed.^[15]^ Moreover, a study conducted in 11 nursing homes in Japan demonstrated that oral care significantly prevented the onset of aspiration and pneumonia, and reduced the number of pneumonitis-related deaths.^[16]^ These results were preliminary, but the report suggested that diseases related to oral/dental conditions could be prevented or improved by promoting oral care among nursing home residents. This means that studies that focus on correlations between dental conditions and diseases in the elderly population who require care, such as nursing home residents, are relatively more important. In addition to these, several previous studies have suggested associations and correlations between the dental conditions of elderly individuals living in nursing homes and their comorbidities.^[17,18]^ To accumulate literature for use in clinical practice, studies in various settings and comorbidities are important.

The aim of the present study was to investigate the correlation between dental conditions and comorbidities in an elderly population using a database constructed from data obtained from nursing homes in Japan.

## 2 Methods

### 2.1 Study design

In this study, we used a database constructed using data obtained from 12 nursing homes consisting of 1008 individuals in Japan. The database was developed and provided by the Health, Clinic, and Education Information Evaluation Institute (HCEI; Kyoto, Japan), which is a general incorporated association. The registration period was from January 1, 2014, to December 31, 2015, and individuals were consecutively included in the database. The data were retrospectively collected from the nursing homes. The database included the sociodemographic/clinical data [age, sex, location before admission, reasons for admission, level of care required, comorbidities, oral condition (number of present, decayed, and filled teeth), use of dentures] of the elderly individuals.

### 2.2 Study population

Individuals with dental and other medical records of nursing homes [sociodemographic/clinical data) (age, sex, location before admission, reasons for admission, level of care required, comorbidities, oral condition (number of present, decayed, and filled teeth), use of dentures] were included in the analysis. After excluding individuals with missing data, the association between the dental condition, comorbidities, and other sociodemographic/clinical factors was analyzed. To maximize the generalizability of the study results, other exclusion criteria were not set.

The following sociodemographic/clinical data were obtained at the time of admission to the nursing home: age, sex, reason for admission, residence before admission (home or hospital), care needs level, dental conditions (number of present teeth, decayed teeth, and filled teeth), and comorbidities (dementia, stroke, bone fracture, arthritis, heart disease, and other common diseases associated with care).

### 2.3 Dental conditions and care needs level

The condition of the teeth was categorized as follows: normal, decayed, and filled. Normal teeth were defined as teeth that were not decayed or filled. According to the World Health Organization criteria, a retained root was considered as a decayed tooth.^[19]^

The care needs level was determined by the local government in Japan, and ranged from 1 (lightest) to 5 (heaviest).^[20,21]^ The process of determination of the level is nationally standardized and includes a visit to the individual's home or hospital by a trained local government official. The computerized algorithm was preliminarily used to determine the level based on 74 items related to physical and mental status. Subsequently, a local committee of specialists determined the level according to the preliminary results and a report of the 12 items of behavioral status by an attending physician. Details were reported by Tsutsui and Muramatsu,^[20]^ but briefly, level 1 means that the individual requires partial help for daily life, and level 5 means that the individual requires full help for daily life.

### 2.4 Statistical analysis

Continuous variables are expressed as mean ± standard deviation (SD), while categorical variables are described as number and percentage (%). The number of teeth (including decayed and filled) across age groups was compared using 1-way analysis of variance. Linear regression models were used to analyze univariate and multivariate associations between the dental conditions, comorbidities, and other sociodemographic/clinical backgrounds.^[22]^ In the multivariate analyses of age, sex and the care needs level were considered potential confounding variables because of their medical implications. The fit of the model was evaluated by the root mean squared error (RMSE). In the supplementary analysis, the prevalence of comorbidities with or without decayed teeth were compared using Fisher exact test. The 2-sided significant level was set at *P* < .05. All statistical analyses were performed using SAS version 9.4 for Windows (SAS Institute Inc., Cary, NC) and Stata 15 (StataCorp, College Station, TX).

### 2.5 Ethical considerations

This study was conducted in accordance with the Declaration of Helsinki and the Ethical Guidelines for Medical and Health Research Involving Human Subjects of the Ministry of Health, Labour and Welfare, Japan. The study protocol was approved by the Ethics Committee of Kyoto University Graduate School and Faculty of Medicine (No. R0811, October 26, 2016). The need for additional informed consent was waived by the committee according to the guidelines.

## 3 Results

### 3.1 Socio-demographic/clinical characteristics of the study subjects

**Figure 1:**
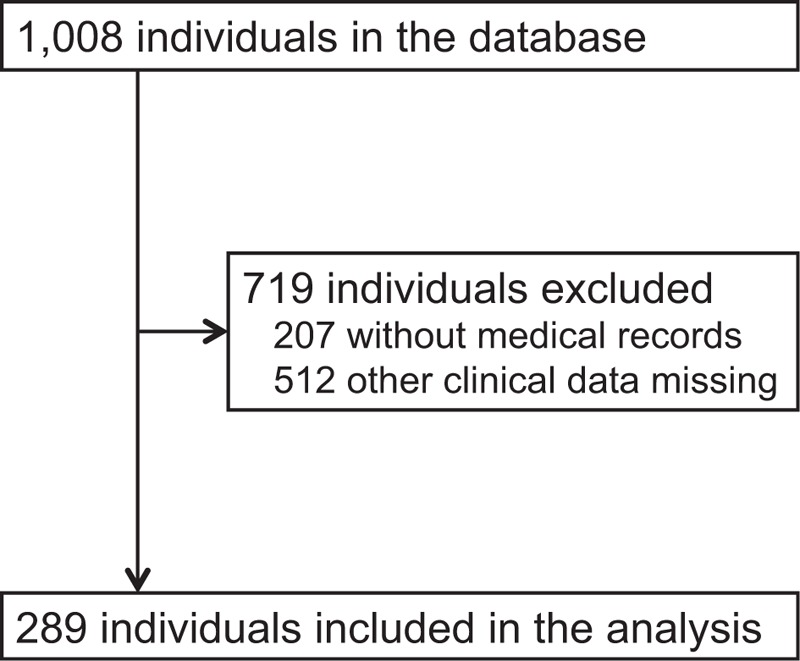
Study flow diagram.

According to the eligibility criteria, 289 elderly individuals [107 men and 182 women; mean age (range), 85 (53–103) years] were included in the analysis (Fig. 1). The reasons for admission (N, %) were as follows: improvement in activities of daily living (266, 92.0%), maintenance of physical/mental function (240, 83.0%), prevention of the progression of complications of dementia (203, 70.2%), caregivers’ burden/distress (158, 54.7%), preparation for going back home (111, 38.4%), and absence of caregivers (59, 20.4%). Several individuals had one or more reasons. Other sociodemographic/clinical characteristics of these individuals are summarized in Table 1. Approximately half of the individuals lived at home before admission. Care level 2 (75, 26.0%) was most frequently required, and 5 (36, 12.5%) most infrequently. Dementia was the most common comorbidity reported (116, 40.1%).

**Table 1:**
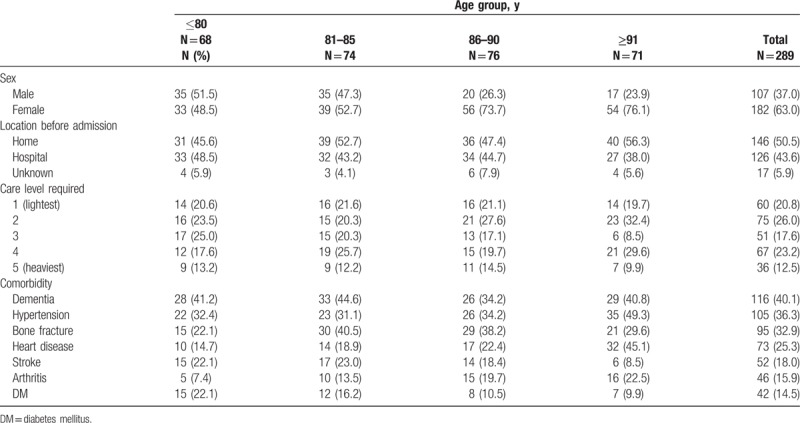
Sociodemographic/clinical characteristics of the study subjects.

### 3.2 Dental health status

The dental health status of the elderly individuals is summarized in Table 2. The number of present teeth among all individuals (mean ± SD) was 11.6 ± 9.6, and decreased with older age [16.2 ± 9.6 (≤80 years), 12.1 ± 8.9 (81–85 years), 11.9 ± 9.5 (86–90 years), 6.5 ± 8.1 (≥91 years); *P* < .001]. Dental conditions such as number of decayed teeth, filled teeth (treated teeth), and denture use are also presented. Among the total population of 289 individuals, the mean number ± SD of decayed teeth was 1.4 ± 3.0 and 4.9 ± 5.2 for filled teeth. Over half of the individuals used dentures (172, 59.5%).

**Table 2:**
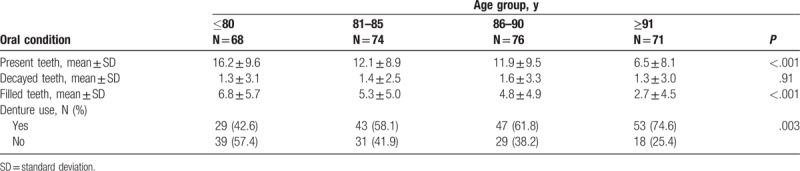
Oral conditions in each age group.

### 3.3 Univariate and multivariate linear regression

Associations between dental status and other sociodemographic/clinical characteristics was analyzed using linear regression models. In the univariate analysis, there was no significant association between the number of present teeth and the following characteristics: sex, residence before admission, and care level required (Table 3). However, the presence of hypertension, heart disease, and arthritis was significantly correlated with the number of present teeth (*P* = .035, .005, and .023, respectively). The presence of heart disease and arthritis was also significantly correlated with the number of normal teeth (*P* = .014 and .034, respectively). Dementia was not significantly associated with the number of present (*P* = .56) and normal teeth (*P* = .78), but instead with the number of decayed teeth (*P* = .018, RMSE = 2.959). In the multivariate regression analysis on comorbidities, the number of decayed teeth was significantly correlated with the number of decayed teeth even after adjusting for confounding variables (*P* = .030, RMSE = 2.959; Table 4). The prevalence of dementia was also significantly higher among the dentate individuals with more than 1 decayed teeth than in individuals without decayed teeth (*P* = .044, Supplementary Table 1).

**Table 3:**
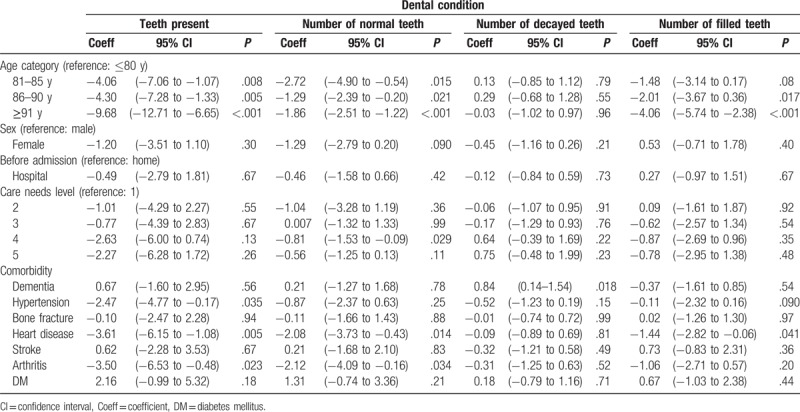
Univariate linear regression analysis of the association between dental conditions and backgrounds.

**Table 4:**
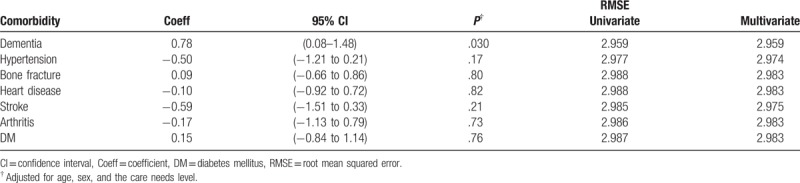
Multivariate linear regression analysis of the association between number of decayed teeth and comorbidities.

## 4 Discussion

The present study investigated the associations between dental conditions and comorbidities in an elderly population living in nursing homes. The number of decayed teeth was significantly correlated with dementia, and this correlation was observed even after adjusting for confounding variables (age, sex, and care needs level). Moreover, the number of treated teeth (filled teeth) was not associated with dementia. These results suggest that dental health could represent a marker of impending dementia, and probably represent a marker of general health status.

In this study population, 107 residents (46.3% of all dentate residents) had at least 1 untreated decay. The incidence of dental decay is increasing in the elderly population.^[23]^ The North American epidemiological studies indicated an association between the presence of root caries (decay) and aging.^[24]^ In addition, a community-based study conducted in India claimed that the prevalence of dental decay in the population aged ≥60 years was > 90%.^[25]^ Previous literature, as well as the current results, indicate that dental decay is problematic in the elderly population, and periodic dental care may have a positive impact on health status. We also focused on other comorbidities. Of these, hypertension, heart disease, and arthritis were significantly associated with the number of present and normal teeth. These findings are consistent with those reported in previously published literature^[10,12,13,26]^; therefore, our results may have generalizability.

However, there are several limitations of this study. The most important limitation was the cross-sectional setting, owing to which causality cannot be inferred using the present results. A study reported by Gil-Montoya et al^[27]^ demonstrated that daily oral hygiene was independently associated with cognitive impairment. Elderly individuals with dementia had difficulty maintaining adequate oral hygiene, and often presented with bacterial dental plaque accumulation and gingival inflammation.^[27]^ In addition, individuals with dementia are less likely to complain about their oral condition, and a lack of access to professional dental care makes it difficult to detect and treat dental decay adequately. The correlations between oral health status and cognitive impairment are still unclear,^[28]^ but even if our findings indicate a reverse-causality, periodic dental care in the elderly population should be prioritized. The limited information included in the database was also a potential limitation. There were no data related to oral health such as issues with nutrition, food consumption, tobacco use, and alcohol consumption.^[29–31]^ The data used in this study were obtained from 12 nursing homes, but only 289 individuals were included in the analysis. Therefore, representativeness of the study sample might be limited. Additional investigations including these aspects may provide more beneficial results in clinical practice; nonetheless, our exploratory results provide valuable suggestions for future research on oral and systemic health status.

In summary, the number of decayed teeth was correlated with dementia. While causality cannot be inferred from the present results, periodic dental examinations, care, and treatments should be prioritized to slow the onset of age-related diseases such as dementia in the elderly population. Additional longitudinal studies are highly desirable to understand the causal relationships between dental conditions and systemic comorbidities.

## Acknowledgment

The authors would like to thank the staff members of the nursing homes and HCEI (Kyoto, Japan) for preparing and providing the data for this study.

## Author contributions

**Conceptualization:** Kazuki Ide, Kahori Seto, Tomoko Usui, Sachiko Tanaka, Koji Kawakami.

**Data curation:** Kazuki Ide, Kahori Seto, Tomoko Usui, Sachiko Tanaka, Koji Kawakami.

**Formal analysis:** Kazuki Ide, Kahori Seto, Sachiko Tanaka.

**Funding acquisition:** Koji Kawakami.

**Investigation:** Kazuki Ide, Kahori Seto, Tomoko Usui, Sachiko Tanaka, Koji Kawakami.

**Methodology:** Kazuki Ide, Kahori Seto, Tomoko Usui, Sachiko Tanaka.

**Project administration:** Kazuki Ide, Kahori Seto, Koji Kawakami.

**Supervision:** Koji Kawakami.

**Validation:** Kahori Seto, Tomoko Usui, Sachiko Tanaka.

**Writing – original draft:** Kazuki Ide, Kahori Seto, Tomoko Usui, Sachiko Tanaka, Koji Kawakami.

**Writing – review & editing:** Kazuki Ide.

## Supplementary Material

Supplemental Digital Content
