# Supplementary material for: Correlation between dental conditions and comorbidities in an elderly Japanese population: A cross-sectional study
Source: Medicine (Baltimore). 2018 Jun 15;97(24):e11075. doi: 10.1097/MD.0000000000011075 (PMC6023670; doi:10.1097/MD.0000000000011075)
Supplement: Supplemental Digital Content [file medi-97-e11075-s001.docx]

**Supplementary Information**

**Supplementary Table 1.** Comorbidities with or without decayed teeth

|  | Decayed teeth | | *P* |
| --- | --- | --- | --- |
|  | - | + |  |
| Comorbidity, n (%) |  |  |  |
| Dementia | 43 (34.7) | 52 (48.6) | 0.044 |
| Hypertension | 47 (37.9) | 31 (29.0) | 0.17 |
| Bone fracture | 48 (38.7) | 29 (27.1) | 0.070 |
| Heart disease | 31 (25.0) | 22 (20.6) | 0.44 |
| Stroke | 26 (21.0) | 15 14.0) | 0.23 |
| Arthritis | 16 (12.9) | 15 (14.0) | 0.85 |
| DM | 15 (12.1) | 17 (16.0) | 0.45 |

DM, diabetes mellitus; *P*, *P*-value.
